# Synthesis of Isoamyl Fatty Acid Ester, a Flavor Compound, by Immobilized *Rhodococcus* Cutinase

**DOI:** 10.4014/jmb.2402.02033

**Published:** 2024-04-19

**Authors:** Ye Won Jeon, Ha Min Song, Ka Yeong Lee, Yeong A Kim, Hyung Kwoun Kim

**Affiliations:** Division of Biotechnology, The Catholic University of Korea, Bucheon 14662, Republic of Korea

**Keywords:** *Rhodococcus* cutinase, isoamyl fatty acid ester, flavor, esterification, docking model

## Abstract

Isoamyl fatty acid esters (IAFEs) are widely used as fruity flavor compounds in the food industry. In this study, various IAFEs were synthesized from isoamyl alcohol and various fatty acids using a cutinase enzyme (Rcut) derived from *Rhodococcus* bacteria. Rcut was immobilized on methacrylate divinylbenzene beads and used to synthesize isoamyl acetate, butyrate, hexanoate, octanoate, and decanoate. Among them, Rcut synthesized isoamyl butyrate (IAB) most efficiently. Docking model studies showed that butyric acid was the most suitable substrate in terms of binding energy and distance from the active site serine (Ser114) γ-oxygen. Up to 250 mM of IAB was synthesized by adjusting reaction conditions such as substrate concentration, reaction temperature, and reaction time. When the enzyme reaction was performed by reusing the immobilized enzyme, the enzyme activity was maintained at least six times. These results demonstrate that the immobilized Rcut enzyme can be used in the food industry to synthesize a variety of fruity flavor compounds, including IAB.

## Introduction

Cutinase is an enzyme produced mainly by plant pathogenic fungi. It is a carboxylic acid ester hydrolase that hydrolyzes the lipid polymer called cutin on the surface of a plant [1, 2]. Cutinase can decompose esters, polyesters, and triacylglycerol in an aqueous environment and perform esterification and transesterification reactions in a non-aqueous environment [3-6]. In this respect, cutinase has similar reactivity to lipase or esterase [2, 7]. Cutinase features a Ser-His-Asp catalytic triad; however, it lacks a hydrophobic cap that typically covers the active site serine. As a result, it exhibits an affinity for a diverse array of substrates beyond cutin. Notably, it stands out as the smallest member among α/β hydrolases [8, 9].

To date, researchers have investigated the structures of fungal cutinases from *Fusarium solani* pisi [10], *Aspergillus oryzae* [11], *Fusarium oxysporum* [12], *Humicola insolens* [13], *Trichoderma reesei* [14], and *Malbranchea cinnamomea* [15]. In addition, bacterial cutinases from *Thermobifida alba* AHK119 [16] and *Thermobifida fusca* [17] have been studied. Ester synthesis reactions by cutinases from *F. solani* pisi and *Burkholderia cepacia* have been explored. Furthermore, investigations have revealed the high enantioselective activity of cutinases from *A. oryzae* and *H. insolens* [18].

Recently, *Rhodococcus* sp. isolated from the Antarctic Ross Sea has been reported to produce cutinase enzyme (Rcut) [19]. The sequence of Rcut gene had 651 nucleotides. It encodes 216 amino acids. It has a signal sequence consisting of 29 amino acids. Structural modeling has revealed that the active site of this enzyme has a catalytic triad of Ser^114^-His^194^-Asp^181^ without a lid structure. Furthermore, studies have reported that Rcut can synthesize short-chain alkyl butyrates using butyric acid and alcohols of varying chain lengths [5].

Short-chain flavor esters are composed of fatty acids and fatty alcohols with chains of about 10 or less carbon atoms [20]. These are used in various food, cosmetics, and pharmaceutical industries [21, 22]. For example, isoamyl esters are synthetic flavoring agents that are stable, colorless liquids with a strong fruity flavor. They are included in fruits such as pineapple, raspberry, strawberry, apricot, banana, pear, and apple. The chemical synthesis employed for producing food ester compounds often occurs at elevated temperatures with the aid of acid or base catalysts. Such high-temperature conditions necessitate extra processing steps and incur additional costs. In contrast, the enzyme-catalyzed approach offers the advantage of conducting reactions under milder conditions, ensuring superior product quality and purity. This method operates effectively at lower temperatures (30–70°C) and reduced pressure, yielding a product that is pure, colorless, and odorless. Advancements in microbiology and fermentation technologies have led to decreased enzyme expenses, while enzyme immobilization techniques have rendered enzymes reusable [9, 22].

In this study, high value-added flavor esters used in the food industry were enzymatically produced using Rcut. For that Rcut was produced using *E. coli* Rosetta gami 2 (DE3) and immobilized on methacrylate divinylbenzene beads. Various isoamyl fatty acid esters (IAFEs) were synthesized using immobilized enzymes. Substrate specificity was then investigated. Optimal conditions for isoamyl butyrate (IAB) synthesis were investigated and a docking model was constructed to explain substrate specificity.

## Materials and Methods

### Materials

Acetic acid, butyric acid, tributyrin, hexanoic acid, octanoic acid, decanoic acid, cyclohexane, molecular sieve, isoamyl alcohol, isoamyl acetate, isoamyl butyrate, isoamyl hexanoate, isoamyl octanoate, isoamyl decanoate, and ampicillin were purchased from Sigma Aldrich Co. (USA). Methanol was purchased from Merck Chemical Co.(Germany). HPLC grade water was purchased from Samchun Chemicals Co. (Republic of Korea). Acetonitrile was purchased from Duksan Pure Chemicals Co. (Republic of Korea). Methacrylate divinylbenzene beads were purchased from GenoFocus (Republic of Korea). LB broth was purchased from Becton, Dickinson and Co.(Republic of Korea). Isopropyl β-D-thiogalactopyranoside was purchased from Duchefa Biochemie BV Co.(Netherland).

### Cutinase Assay

Cutinase activity was analyzed using p-nitrophenyl caprylate (pNPC) as a substrate [5]. The reaction mixture (1 ml) was composed of 950 μl of 50 mM Tris-HCl (pH 8.0), 40 μl of ethanol, and 10 μl of acetonitrile with 10 mM pNPC. The reaction was carried out by adding the enzyme solution to reaction mixture, followed by incubation at 30°C for 3 min. The absorbance at 405 nm was measured to determine the amount of p-nitrophenol (pNP) produced. One unit of cutinase was defined as the amount of enzyme that producing 1 μmol of pNP per min.

### Preparation of Biocatalysts

*E. coli* BL21 (DE3) (F^-^ *omp*T *hsd*S_B_ (r_B_^-^ m_B_^-^) *gal dcm* (DE3) and *E. coli* Rosetta gami 2 (DE3) (Δ(ara–leu)7697 ΔlacX74 ΔphoA PvuII phoR araD139 ahpC galE galK rpsL (DE3) F’[lac^+^ lacI ^q^ pro] gor522::Tn10 trxB pRARE2 (Cam^R^, Str^R^, Tet^R^)) transformed with the pET22 vector containing the Rcut gene [19] were cultured as follows. First, *E. coli* BL21 (DE3) was inoculated into LB broth (200 ml) supplemented with ampicillin (100 μM). It was then cultured at 37°C with shaking at 210 rpm until the optical density (OD) value at 600 nm reached 0.5. After β-D-thiogalactopyranoside (IPTG) (1 mM) was added, the bacterial culture was further incubated at 20°C for 24 h. After collecting cells through centrifugation (4,000 ×*g*, 10 min, 4°C), cells were resuspended in 10 ml of deionized H_2_O. After disrupting cells using ultrasonic waves, cell lysate was centrifuged (10,000 g, 10 min, 4°C) and upper and lower layers were collected, respectively.

Second, *E. coli* Rosetta gami 2 (DE3) was inoculated into LB broth (200 ml) supplemented with ampicillin (100 μM) and chloramphenicol (100 μM) and cultured with shaking at 37°C until the OD value at 600 nm reached 0.3. The next process was performed in the same manner as the *E. coli* BL21 (DE3) strain. Afterwards, pNPC assay and protein quantification were performed to measure enzyme specific activity. SDS-PAGE was performed to confirm the expression of the enzyme.

*E. coli* Rosetta gami 2 (DE3) strain transformed with the pET22/Rcut vector was cultured to express the Rcut protein. The supernatant of the cell lysate was then immobilized on methacrylate-divinylbenzene (MA-DVB) beads as follows. First, MA-DVB beads (1 g) were added to methanol (10 ml) and washed at 160 rpm for 1 h at 25°C. Methanol was removed and beads were washed with 10 ml of 100 mM potassium phosphate buffer (pH 6.5) for 1 h under the same conditions. After removing the buffer, MA-DVB beads were mixed with 4 ml of cell free extract (CFE) (Rcut equivalent to 106 U), 3.5 ml of deionized H_2_O, 2.5 ml of 2 M ammonium sulfate, and 25 mM glutaraldehyde (total 10 ml) at 4°C. The reaction was performed for 24 h. Afterwards, beads on which the protein was immobilized were washed using potassium phosphate buffer. Afterwards, beads from which the buffer was removed were completely dried using a Speed-Vac and stored at 4°C. The immobilization yield was calculated with the following formula based on the amount of initial protein and the amount of protein remaining in the supernatant after the immobilization process:

Immobilization yield = (P_initial_ – P_unbound_) / P_initial_ * 100 (%) (1)

### Enzymatic Synthesis of IFAE

An esterification reaction was performed using immobilized Rcut (immRcut) (Scheme 1). IAFE synthesis reaction was performed using isoamyl alcohol (IAOH) and fatty acid as substrates. For fatty acids, acetic, butyric, hexanoic, octanoic, and decanoic acid were used to investigate substrate specificity of enzymes according to the number of carbon chains. After 100 mM IAOH, 100 mM fatty acid, 70 mg ImmRcut, and 100 mg molecular sieve were added to 5 ml cyclohexane, the reaction was performed at 30°C in a shaking incubator at 210 rpm for 5 h. The reaction solution was analyzed through HPLC. The HPLC peak area was compared to the standard curve of the standard material to measure the amount of product (Fig. S1).

Esterification and transesterification reactions were performed using immRcut. Briefly, 70 mg immRcut and 100 mg molecular sieve were added to 5 ml cyclohexane. IAOH and BA were used as substrates for the esterification reaction. IAOH and tributyrin (TBN) were used for the transesterification reaction. The reaction was conducted for a total of 24 h. Other conditions were the same as the previous method. After the 24 h reaction, the reaction mixture was analyzed through HPLC.


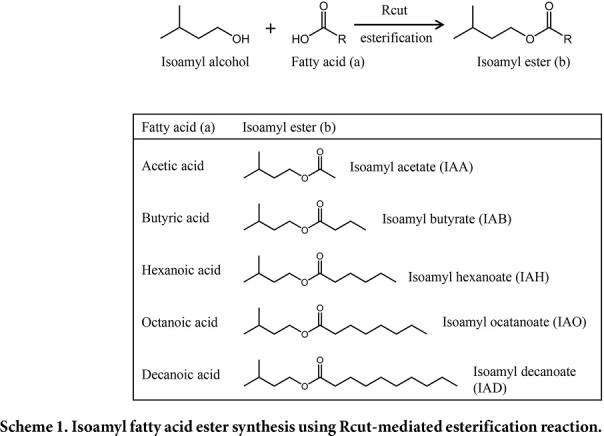


### HPLC Analysis

The substrate and product contained in the reaction mixture were assayed using Agilent 1100 series HPLC. A Cogent Bidentate C18 column (4.6 mm × 250 mm, 5 μm particle size; microSolv Technology Corp., USA) was used. As a mobile phase, acetonitrile : water = 9:1 was used. The flow rate was set at 1.0 ml/min and the temperature was set at 35°C. A refractive index detector was used to analyze isoamyl esters. The temperature was set at 35°C.

### Optimization of Enzyme Reaction

The reaction was performed while increasing the concentration of each substrate. After 70 mg immRcut and 100 mg molecular sieve were added to 5 ml cyclohexane, the enzymatic reaction was performed while increasing IAOH and BA (or TBN) from 50 mM to 500 mM. The reaction was performed in the same manner as previously described.

The IAOH concentration was fixed at 300 mM. BA was added at 300 mM, 400 mM, or 500 mM. The reaction was performed in the same manner as previously described. Additionally, BA concentration was fixed at 300 mM and IAOH was added at 300 mM, 400 mM, or 500 mM. The reaction was performed in the same manner as previously described.

To find the optimal temperature for the enzyme reaction, the IAB synthesis reaction was performed while varying the reaction temperature from 20 to 60°C. After 300 mM IAOH, 300 mM BA, 70 mg immRcut, and 100 mg molecular sieve were added to 5 ml cyclohexane, the reaction was performed for 6 h or 24 h. In order to check the amount of IAB produced according to the reaction time, the amount of IAB synthesized was measured during reaction. After 300 mM IAOH, 300 mM BA, 70 mg immRcut, and 100 mg molecular sieve were added to 5 ml cyclohexane, the reaction was performed at 30°C for 20 h to analyze the amount of IAB.

### Repeated use of ImmRcut Enzyme

The feasibility of repeated use of the immRcut was investigated. First, 300 mM IAOH, 300 mM BA, 70 mg immRcut, and 100 mg molecular sieve were added to 5 ml cyclohexane. The reaction was performed at 30°C with shaking at 210 rpm. After 24 h of reaction, the mixture solution was removed and beads were washed with cyclohexane. Afterwards, cyclohexane, IAOH, and BA were added and enzyme synthesis reaction was performed for 24 h. It was repeated six times. The mixed solution after reaction was analyzed through HPLC. Additionally, excluding molecular sieve, 300 mM IAOH, 300 mM BA, and 70 mg immRcut were added to 5 ml cyclohexane. Other than that, the reaction was performed in the same manner as described above.

### In silico Molecular Docking Analysis Using Autodock

Autodock 4.2.6 and MGLTools 1.5.7 version (https://ccsb.scripps.edu/) were used for molecular docking analysis. A homology structure model of Rcut was made with *F. solani* cutinase (PDB: 1CEX) as a template using amino acid sequences of *R*cut. 3D modeling was performed in SWISS-MODEL (https://swissmodel.expasy.org/). 3D models of IAOH, acetic acid, butyric acid, hexanoic acid, octanoic acid, and decanoic acid were downloaded from https://pubchem.ncbi.nlm.nih.gov/. Protein-ligand molecule docking was performed according to the tutorial of AutodockTools. Grid box coordinates were: X, 7.5; Y, 55.8; and Z, 17.5. Spacing (Å) was set to be 0.250. Genetic algorithm parameters were set as basic settings (number of GA run: 10; population size: 150). The docking output was set with Lamarcarkian GA of Autodoc 4.2.

## Results and Discussion

### Preparation of Biocatalysts

**Fig. 1:**
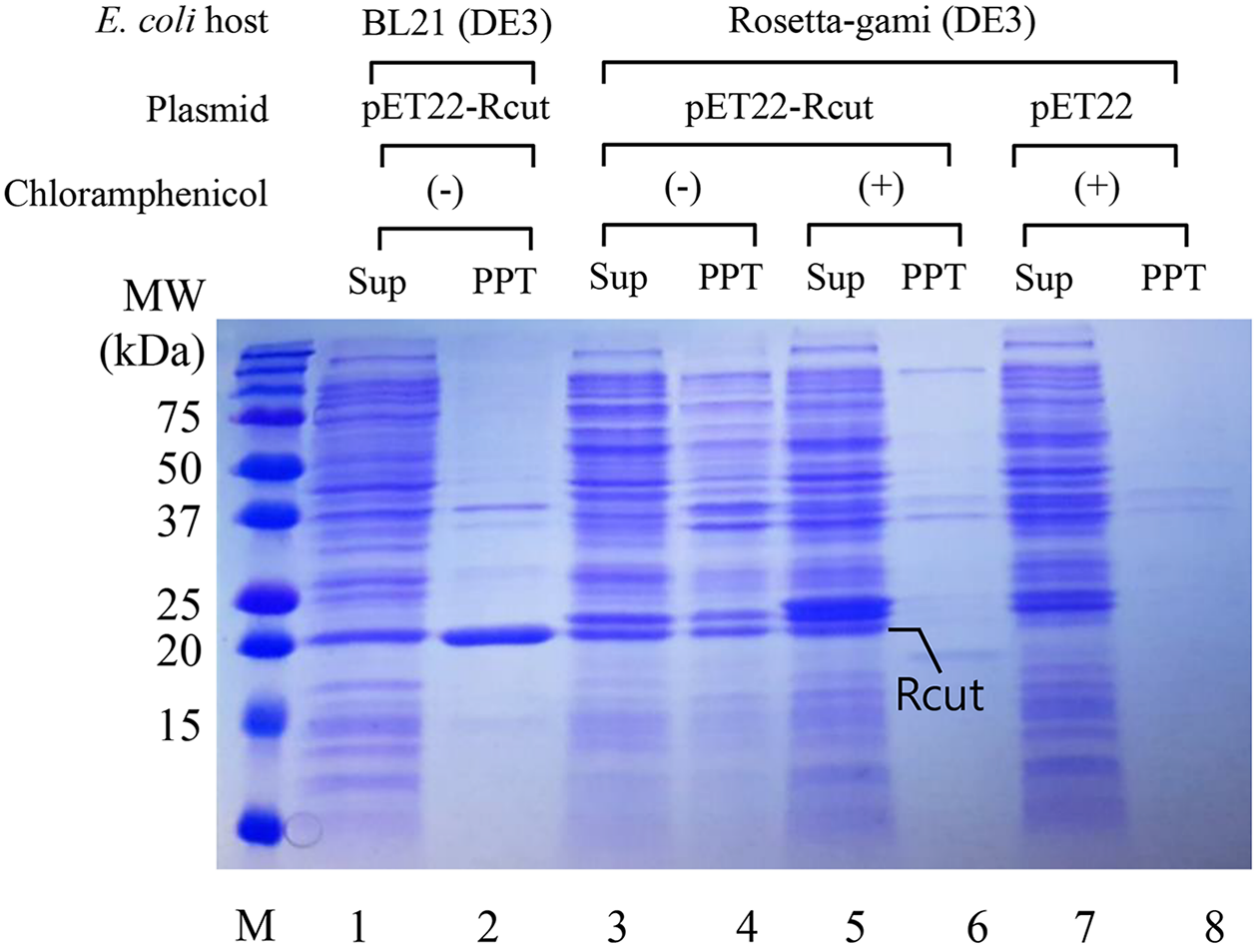
Expression of *Rhodococcus* cutinase in *E. coli* cells. SDS-PAGE was performed using supernatant and precipitate after *E. coli* cells were lysed. Lane M, size marker; Lanes 1–2, *E. coli* BL21(DE3); Lanes 3–8, *E. coli* Rosetta-gami (DE3); Lanes 1–6, pET22-Rcut; Lanes 7–8, pET22; Lanes 1–4, no chloramphenicol; Lanes 5–8, chloramphenicol.

To evaluate the expression of Rcut, the pET22-Rcut plasmid was introduced into *E. coli* BL21 (DE3) and cultured. Following cell lysis, the resulting lysates were centrifuged, producing supernatant and precipitate fractions for analysis via SDS-PAGE (Fig. 1, lanes 1 and 2). The presence of Rcut was observed as a distinct 20 kDa band. Notably, the precipitate exhibited a higher abundance of the Rcut protein compared to the supernatant.

In an alternative expression approach, *E. coli* Rosetta gami 2 (DE3) was employed as the host strain for Rcut expression. *E. coli* Rosetta gami 2 (DE3) harbors mutations in the trxB and gor genes, which encode enzymes responsible for breaking intracellular disulfide bonds. Transformation with the pET22-Rcut plasmid was conducted, followed by SDS-PAGE analysis of the resulting cell lysatés supernatant (Fig. 1, lane 5) and precipitate (Fig. 1, lane 6). The intensities of the Rcut bands observed in lanes 3 and 5 of Fig. 1 appeared to be similar.

Enzyme activity was quantified as follows: 91 U/mg for *E. coli* BL21, 106 U/mg for *E. coli* Rosetta gami chloramphenicol (+), and 65 U/mg for *E. coli* Rosetta gami chloramphenicol (-). Notably, Rcut activity was highest in *E. coli* Rosetta gami chloramphenicol (+). This increased expression of Rcut, a protein featuring two disulfide bonds, was attributed to the inactivation of the trxB and gor genes in this particular strain.

Proteins in the supernatant were immobilized on MA-DVB beads. The immobilization yield was calculated by measuring the amount of initial protein and the amount of the remaining protein in the buffer after the immobilization process [5, 23]. The immobilization yield was measured to be about 97%, which showed that almost all proteins in CFE bound to MA-DVB beads (Table S1). Afterwards, we performed IAFE synthesis using these Rcut-immobilized beads (immRcut).

### Esterification Using immRcut

Several research groups have performed ester synthesis using cutinase. According to their results, the synthesis efficiency differed depending on the length of the substrate. *F. solani* *pisi* cutinase and *B. cepacia* NRRL B 2320 cutinase showed the highest esterification efficiencies when using a C4-C6 substrate [24, 25]. *T. fusca* cutinase had a high esterification yield when C3-C8 acids were used as substrates [26].

**Fig. 2:**
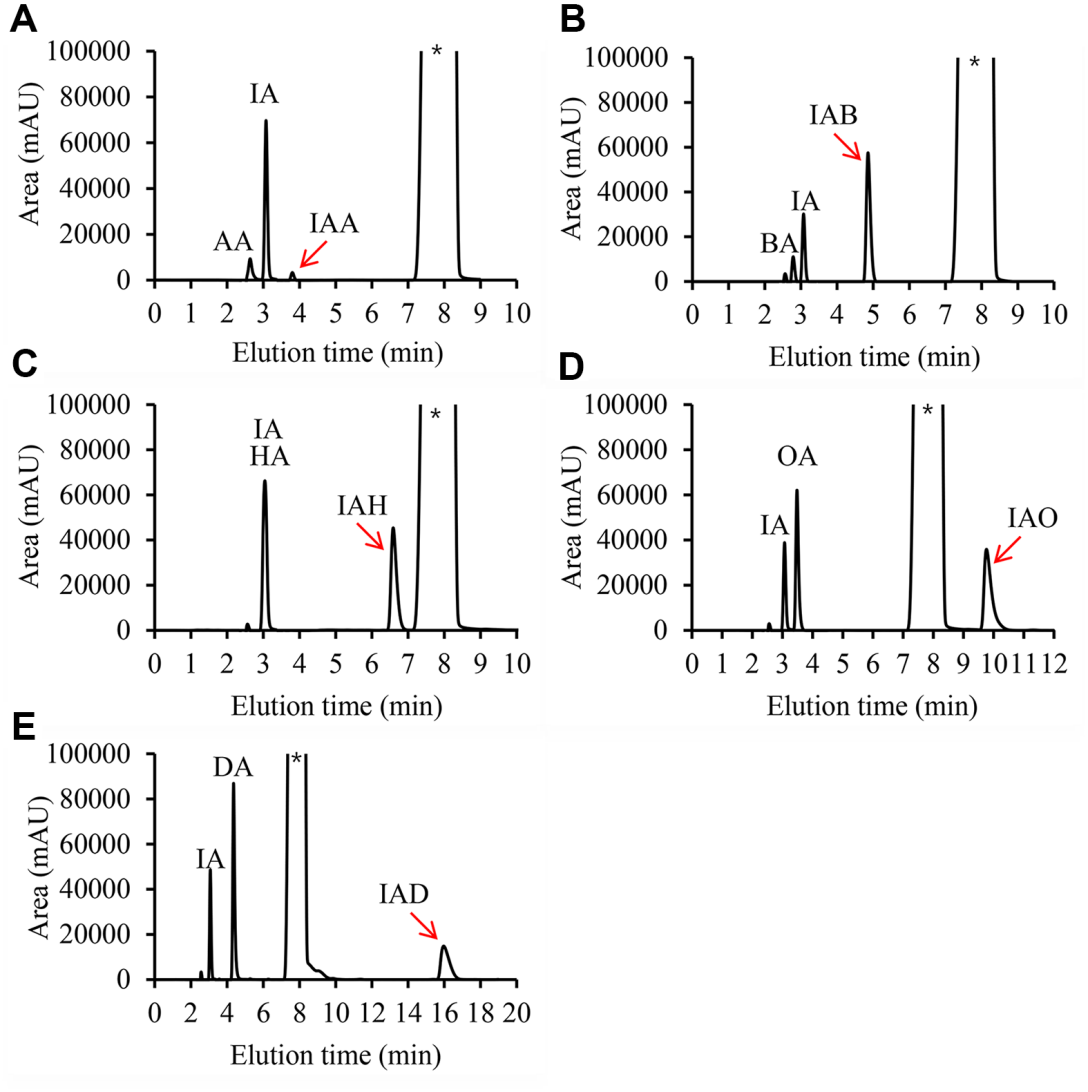
HPLC analysis of reaction products. HPLC was performed after 5 h-reaction using IAOH and various fatty acids. IAOH, isoamyl alcohol; AA, acetic acid; BA, butyric acid; HA, hexanoic acid; OA, octanoic acid; DA, decanoic acid; IAA, isoamyl acetate; IAB, isoamyl butyrate; IAH, isoamyl hexanoate; IAO, isoamyl octanoate; IAD, isoamyl decanoate. *, cyclohexane solvent peak.

In this research, IAFEs were synthesized through an esterification reaction using immRcut (Scheme 1). IAOH (100 mM) and each fatty acid (100 mM) were added to the solvent cyclohexane, respectively, and the reaction product was analyzed through HPLC (Fig. 2). Elution time of each product was found: isoamyl acetate (IAA), 3.6–3.9 min; isoamyl butyrate (IAB), 4.6–5.3 min; isoamyl hexanoate (IAH), 6.5–7 min; isoamyl octanoate (IAO), 9.6–10.3 min; and isoamyl decanoate (IAD), 15.8–16.3 min. Standard curves showing the relationship between molarity and peak area were also constructed (Fig. S1).

**Fig. 3:**
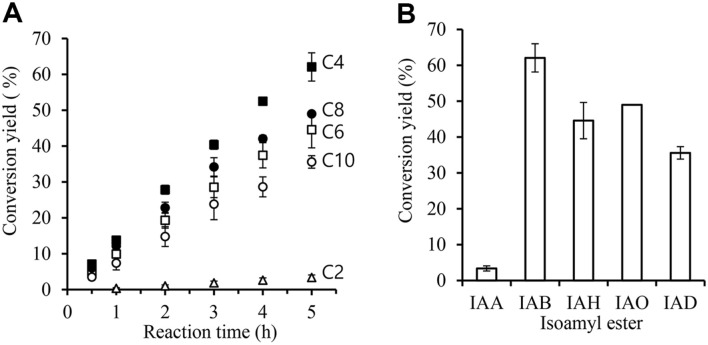
Isoamyl fatty acid ester synthesis using IAOH and various fatty acids. Reaction was carried out with 100 mM of IAOH and 100 mM of fatty acid. The molar concentration was calculated from the product peak area and the conversion yield was calculated. (**A**) Time course of conversion yield was calculated; (**B**) Conversion yield after 5 h-reaction. △, isoamyl acetate (IAA); ■, isoamyl butyrate (IAB); □, isoamyl hexanoate (IAH); ●, isoamyl octanoate (IAO); ○, isoamyl decanoate (IAD).

Fig. 3A shows conversion yields of five IAFEs. Initial conversion rates of IAA, IAB, IAH, IAO, and IAD were 8.57, 192, 136, 163, and 113 mM h^-1^ g^-1^ beads, respectively. This showed that reaction products were quickly synthesized in the following order: IAB, IAO, IAH, IAD, and IAA. When comparing conversion yields after 5 h of reaction, the conversion yield of IAB was the highest at about 62%, while that of isoamyl acetate was the lowest at about 3.4% (Fig. 3B). Therefore, among various fatty acids, BA was found to be the most suitable substrate for Rcut.

### Docking Modeling

**Fig. 4:**
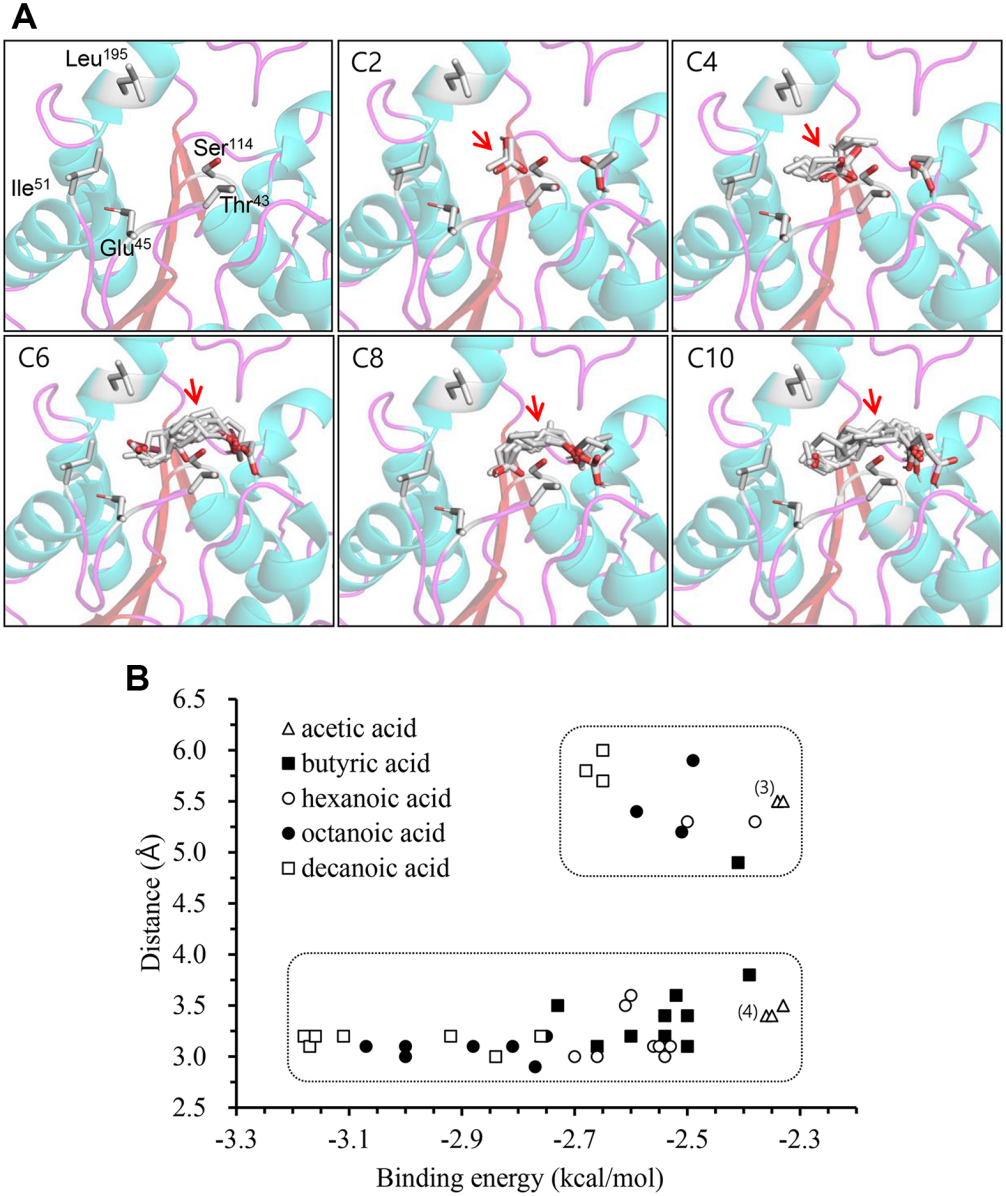
Docking models of each fatty acid at the active site pocket of Rcut enzyme. (**A**) Five amino acids in the Rcut active site and 10 docking candidates of each fatty acid were indicated; (**B**) For 10 candidates of each fatty acid, distances between the O^γ^ of Rcut Ser^114^ and the carbonyl carbon of the fatty acids were measured and binding energies of each candidate were calculated. The numbers (3) and (4) depicted on the graph indicate the distribution of three and four acetic acid candidates, respectively, sharing the same binding energy and distance.

The homology model of Rcut has been already constructed based on *F. solani* cutinase (PDB: 1CEX) as a template [10]. Thus, we created a docking model for Rcut and fatty acids to explain differences in reaction rates depending on the chain length (C_2_–C_10_) of fatty acids. In the ester synthesis process, fatty acid and Ser^114^ first combine to form an acyl enzyme through the 1st tetrahedral intermediate. Then IAOH attacks the carbonyl carbon of acyl group to form the 2nd tetrahedral intermediate. Therefore, substrate specificity is determined from the first half of the acylation reaction. Fortunately, because Rcut did not have a lid structure covering the active site, we were able to create a docking model using Rcut's protein structure. Five amino acids (T43, E45, I51, S114, L195) composed the binding pocket and interacted with the substrate that approached the active site (Fig. 4A).

We performed docking modeling for each fatty acid (C_2_–C_10_) and found 10 of each of the most stable candidates (Fig. 4A). The binding energy of each candidate was calculated. In Fig. 4B, when considering the binding energy represented on the horizontal axis of the graph, the more negative the value, the stronger the binding force. The distance (D) between γ-oxygen of Ser^114^ and carbonyl carbon of candidate was measured and expressed as coordinates (Fig. 4B). In order for the candidate to form an acyl enzyme well, its binding energy should be high and the D value should be close to 3 Å [27]. However, although its binding energy was high, if the D value was too big, this 'interfering candidaté actually could not form an acyl enzyme. It could be seen that there are three interfering candidates for decanoic acid (5.5–6.0 Å), three for octanoic acid (5.0–6.0 Å), and two for hexanoic acid (5.3 Å). On the other hand, there is only one interfering candidate for BA (4.8 Å). Most acetic acids have weak binding energies and four of them have high D values. These results of the docking model confirmed that BA formed the acyl enzyme most effectively. As a result, IAB was formed the fastest.

### Comparison of Esterification and Transesterification

**Fig. 5:**
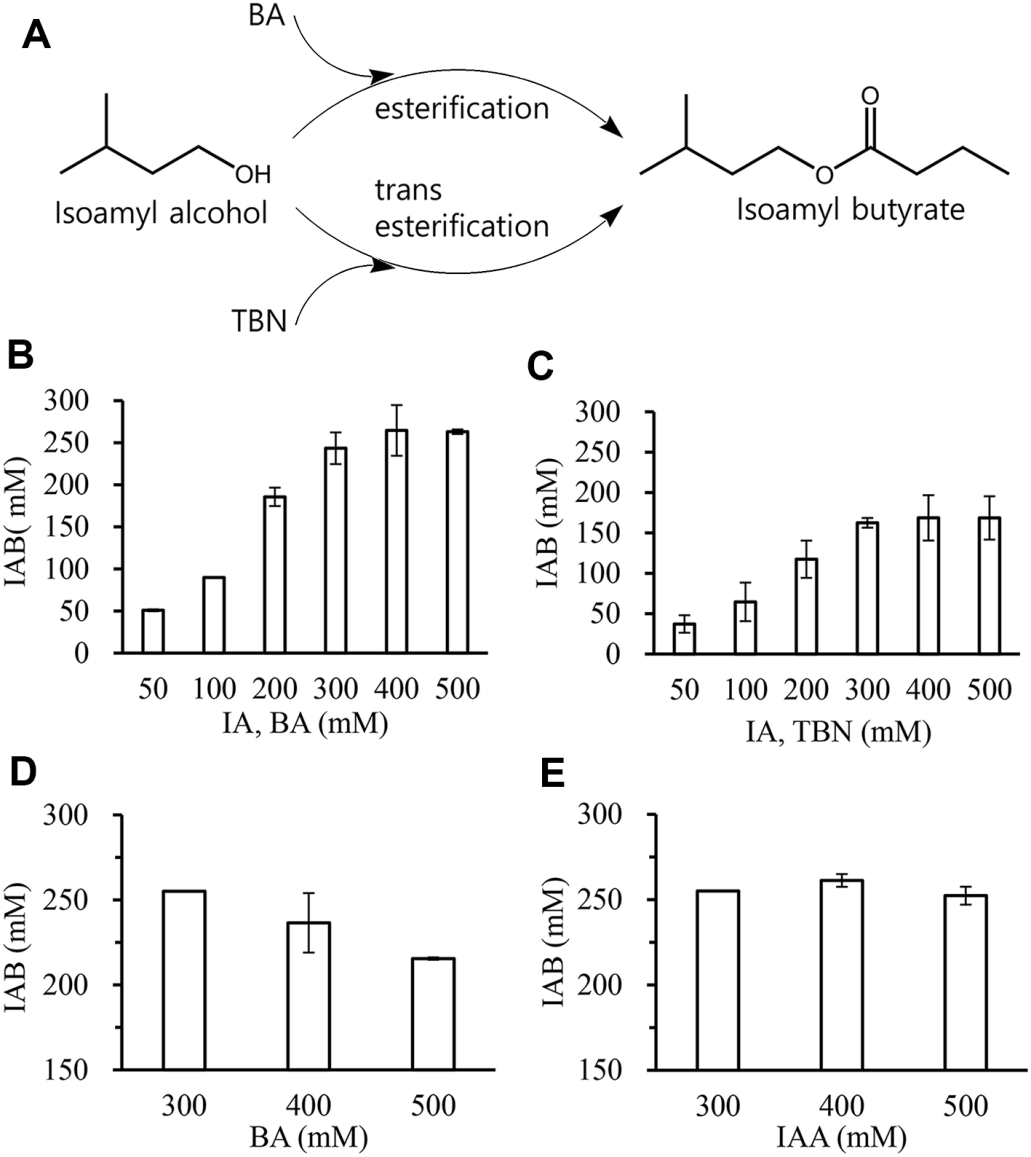
Effects of substrate concentration on conversion yield. (**A**) Isoamyl butyrate was produced by cutinasemediated esterification and transesterification; (**B**) Amount of isoamyl butyrate was measured after 24 h-reaction using butyric acid and IAOH (50–500 mM); (**C**) Amount of isoamyl butyrate was measured in a 24 h-reaction using TBN and IAOH (50–500 mM); (**D**) Amount of isoamyl butyrate was measured while the IAOH concentration was fixed at 300 mM and the butyric acid concentration was increased from 300 to 500 mM; E, Amount of isoamyl butyrate was measured while the butyric acid concentration was fixed at 300 mM and the IAOH concentration was increased from 300 to 500 mM.

Cutinase can perform esterification and transesterification reactions [25, 28]. To determine which reaction was more efficient for IAB synthesis using Rcut, each reaction was performed for 24 h. IAB in the reaction solution was then analyzed by HPLC (Fig. 5). It was found that the esterification reaction yield was relatively higher than the transesterification reaction. For example, when the reaction was performed using 100 mM BA, approximately 89.8 mM of IAB was synthesized (Fig. 5B). When the reaction was performed using 100 mM TBN, approximately 64.6 mM of IAB was synthesized (Fig. 5C). It could be presumed that the rate at which acyl enzyme is formed from BA is faster than that at which it is formed from TBN.

### Optimization of Enzymatic IAFE Synthesis

Meanwhile, the amount of IAB increased as the substrate (IAOH and BA) concentration increased from 50 mM to 300 mM. However, it did not increase further when the substrate concentration exceeded 300 mM. To reveal the reason, an enzyme reaction was performed with different concentrations of each substrate. First, the reaction was performed by adding 300, 400, or 500 mM of BA to 300 mM of IAOH (Fig. 5D). At 300 mM BA, approximately 250 mM IAB was produced. However, as BA concentration increased, the amount of IAB produced decreased. The reaction was then performed by adding 300, 400, or 500 mM of IAOH to a BA concentration of 300 mM. At 300 mM IAOH, approximately 250 mM IAB was produced. A similar amount of IAB was produced even as the IAOH concentration increased (Fig. 5E). These results revealed that enzyme activity was inhibited only when BA exceeded 300 mM.

Loss of enzyme activity can occur due to accumulation of various substances, including water, acids, and alcohols. When Rcut enzyme was immobilized on MA-DVB beads and water was removed, minimal water remained in the vicinity of the enzyme. BA has a pKa value of 4.84. When it is used at a high concentration of 300 mM or more, it is presumed that it can inhibit the activity of the enzyme by lowering the pH of the water around the enzyme. In previous studies, it was observed that in the alkyl ester synthesis using goat pregastric lipase, the catalytic environment shifted with increasing acid concentrations. As the content of fatty acids in the unbuffered organic solvent rose, the pH decreased [29]. Furthermore, investigations into the synthesis of isoamyl acetate utilizing immobilized lipase have indicated that alterations in the pH of the reaction medium due to the presence of reactants or products, particularly if they are bases or acids, can have adverse effects on enzyme activity and reaction rates [30].

**Fig. 6:**
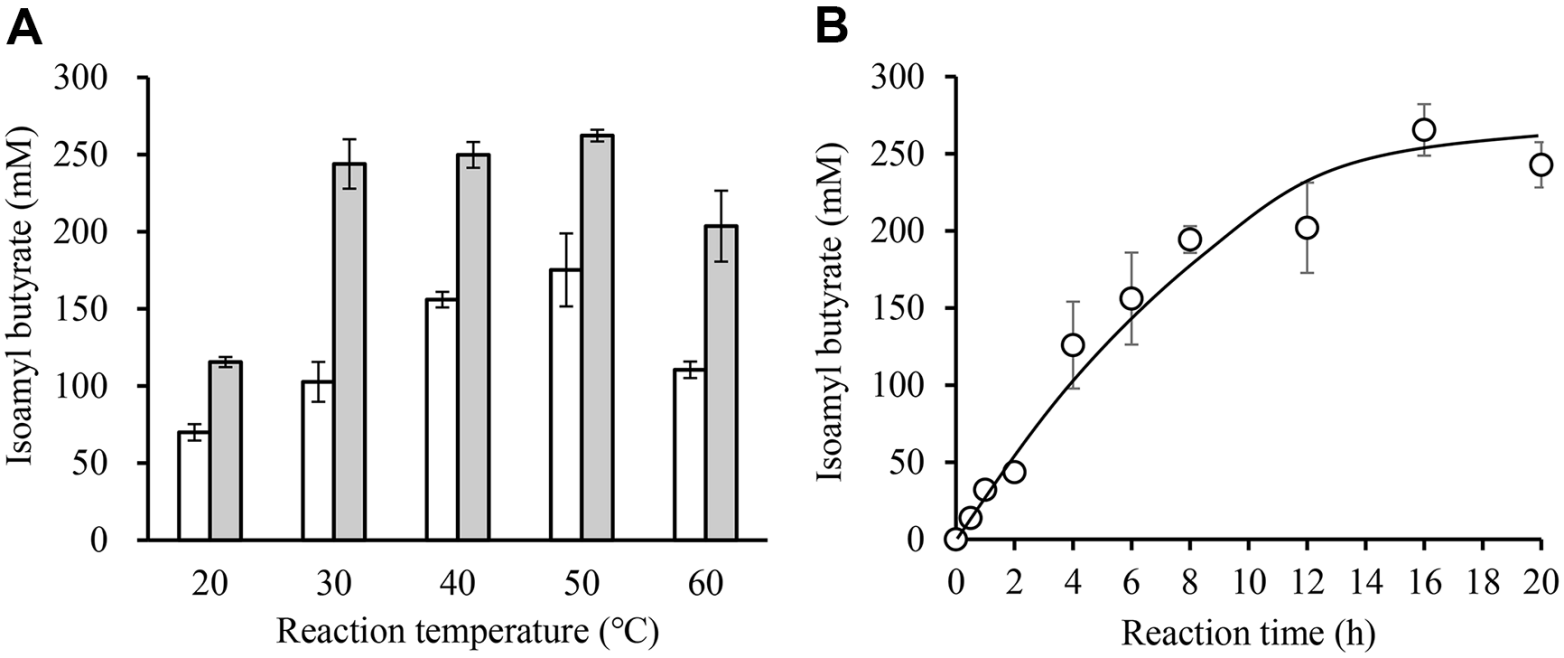
Effects of temperature and reaction time on conversion yield. The enzyme reaction was performed by adding 100 mg molecular sieve, 300 mM IAOH, 300 mM butyric acid, and 70 mg immRcut to 5 ml cyclohexane. (**A**) The reaction was performed at various reaction temperatures (20°C–60°C) and the amount of isoamyl butyrate produced was measured after 6 h and 24 h. (**B**) The reaction was performed at 30°C for up to 20 h and the amount of isoamyl butyrate produced according to the reaction time was measured.

The effect of reaction temperature for the esterification reaction on IAB synthesis using ImmRcut was investigated. The results from the 6-h reaction were analyzed to compare the initial reaction rates, while the results from the 24-h reaction were analyzed to assess the maximum yield of the product. First, after 6-h of reaction, the amount of IAB produced was analyzed by HPLC. When the enzyme reaction was performed at 50°C, 175 mM of IAB was synthesized, showing the highest activity (Fig. 6A). Additionally, the product was analyzed after 24-h to compare the amount of IAB finally synthesized. The concentrations of IAB synthesized at 30, 40, and 50°C were measured to be 243, 249, and 262 mM, respectively. At these temperatures, the IAB concentration was approximately 250 mM, with a production yield of 80%. Because the substrate and reaction products might be unstable at high temperatures for a prolonged reaction process, the reaction temperature was set at 30°C in subsequent experiments.

The amount of IAB produced according to reaction time was analyzed. After 300 mM of each substrate was added, the reaction was performed at 30°C. The amount of IAB produced continued to increase, reaching 200 mM after 12 h. After that, IAB gradually increased. After 250 mM of IAB was synthesized, the amount of IAB no longer increased (Fig. 6B).

### Reusability of Rcut

**Fig. 7:**
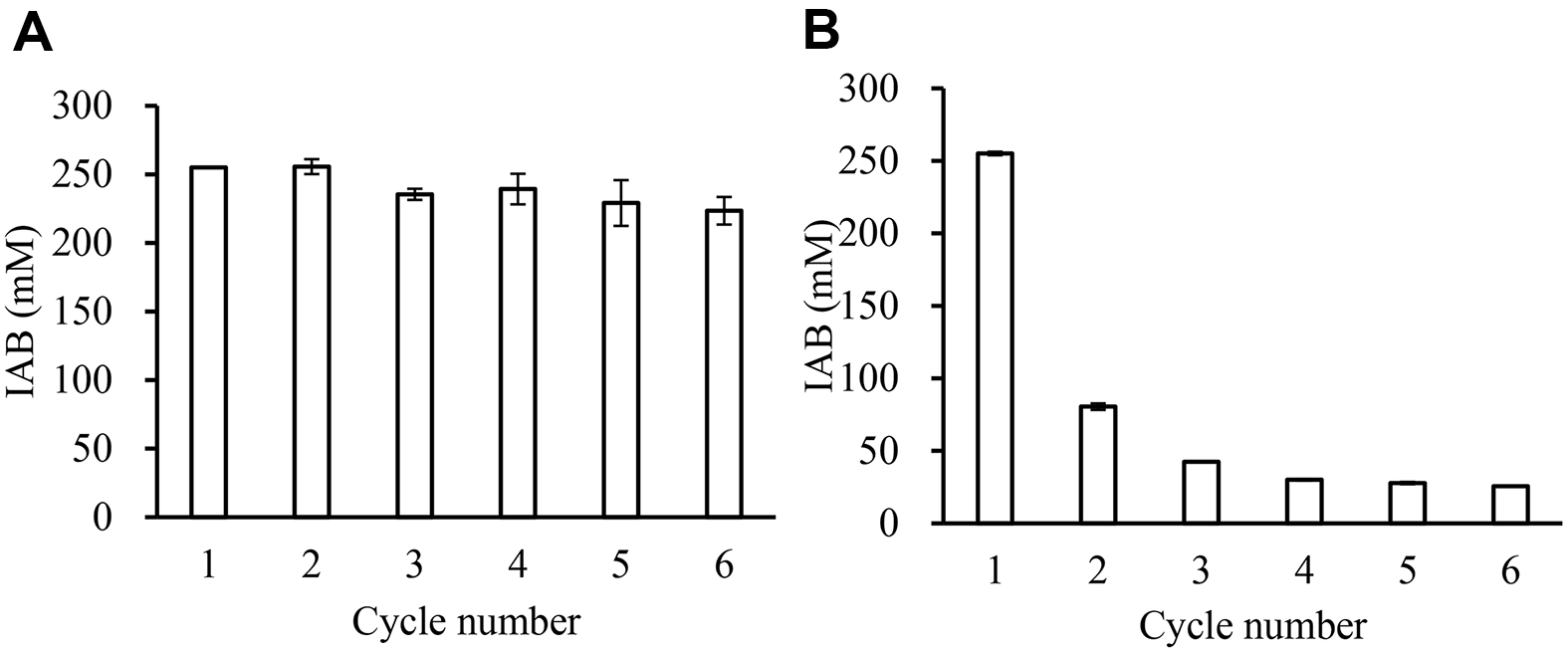
Reusability of immobilized Rcut. The amount of isoamyl butyrate synthesized by immobilized Rcut was measured after a 24 h-reaction. Reaction system contained 300 mM IAOH, butyric acid, and immobilized Rcut in cyclohexane. (**A**) Reaction with a molecular sieve, (**B**) Reaction without a molecular sieve.

To investigate whether immRcut could be reused, esterification reaction was performed several times (Fig. 7). After an enzyme mixture containing the substrate, solvent, and molecular sieve was reacted for 24 h, the mixture was removed. The enzyme and molecular sieve were washed using a solvent. The substrate and solvent were then added again and the reaction was repeated for 24 h for a total of five times. In the enzyme reaction solution containing molecular sieve, approximately 255 mM of IAB was synthesized in the first reaction and approximately 223 mM of IAB was synthesized in the reaction when the enzyme was reused six times (Fig. 7A). In previous studies, it was found that cutinase-CLEA made using *Fusarium* ICT SAC1 cutinase retained over 90% activity after three uses, and it maintained 50% activity after eight uses [31]. Additionally, alkylbutyrate synthesis reactions were conducted by immobilizing cutinase from *F. oxysporum*. After three repeated uses, 80% activity was retained, and after four uses, 54% activity was maintained [28].

In the enzyme reaction solution without a molecular sieve, about 255 mM of IAB was synthesized in the first reaction. However, only about 80 mM of IAB was synthesized in the second reaction and about 26 mM of IAB was synthesized in the sixth and final reaction (Fig. 7B). These results revealed that immRcut activity was maintained only in the enzyme reaction containing a molecular sieve.

In an esterification reaction, water is produced during the reaction process [32]. If a molecular sieve is absent, it is assumed that enzyme activity decreases due to water produced [33, 34]. That is, IAOH and BA are partially soluble in water. They can irreversibly inhibit enzyme activity. BA is a short-chain polar acid. It concentrates in the microaqueous layer and causes a pH drop in the enzyme microenvironment, leading to enzyme inactivation.

Taken together, these results demonstrate that immobilized Rcut can synthesize various IAFEs in non-polar solvents. Among them, IAB, which has a fruity flavor, was synthesized with a high yield. Through a docking model, it was confirmed that Rcut enzyme could specifically bind to BA. Immobilized Rcut was capable of synthesizing 250 mM IAB. It could be used repeatedly more than six times. By enhancing the production of Rcut in *E. coli* and engineering a mutant enzyme capable of retaining its activity even under acidic conditions, it can be employed for the synthesis of diverse fruit-flavored compounds, such as IAB, of significant importance in the food industry.

## Supplemental Materials

Supplementary data for this paper are available on-line only at http://jmb.or.kr.
