# Supplementary material for: Synthesis of Isoamyl Fatty Acid Ester, a Flavor Compound, by Immobilized Rhodococcus Cutinase
Source: J Microbiol Biotechnol. 2024 Apr 19;34(6):1356–64. doi: 10.4014/jmb.2402.02033 (PMC11239401; doi:10.4014/jmb.2402.02033)
Supplement: Supplementary file 1 [file jmb-34-6-1356-supple.pdf]

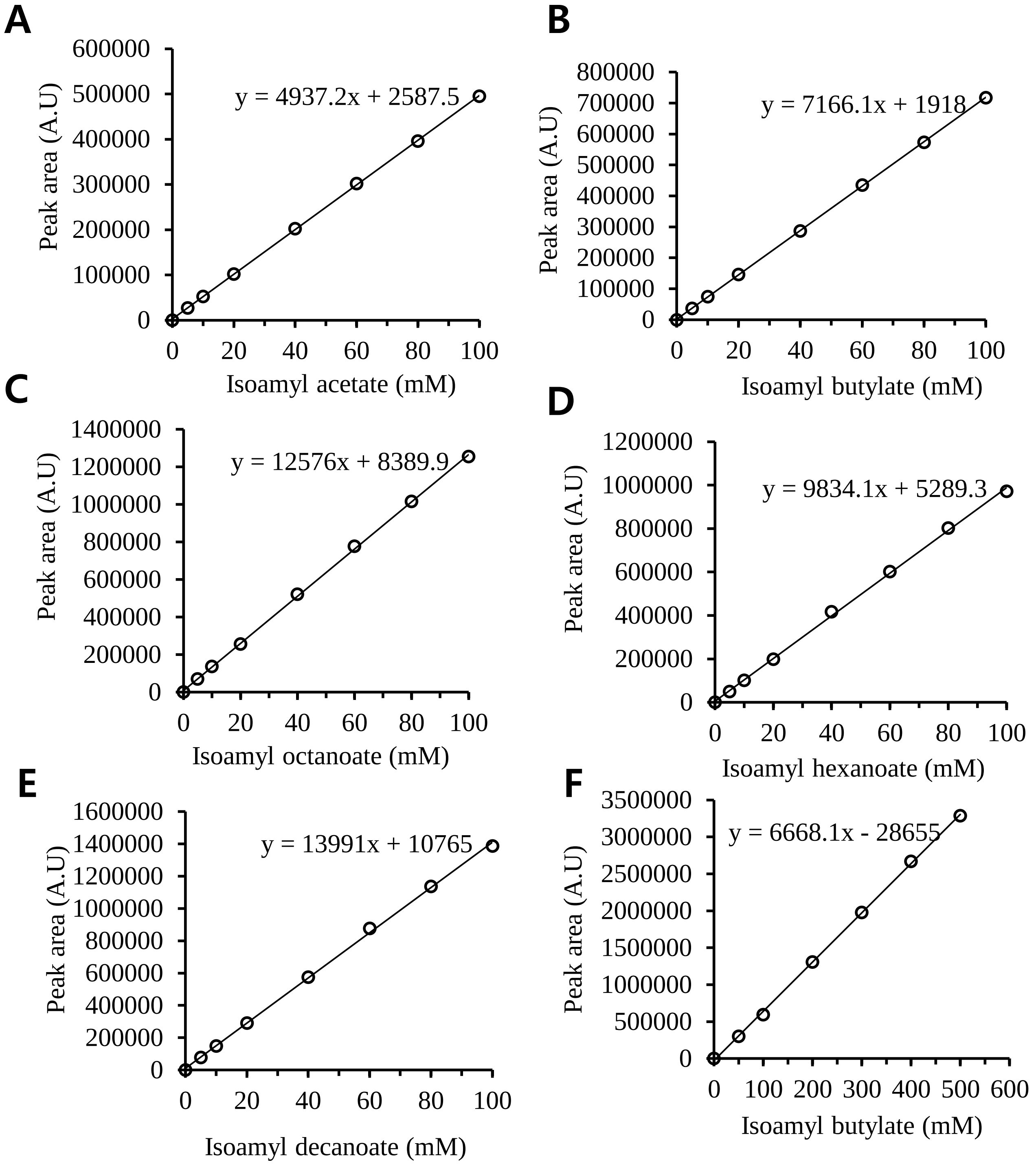

Fig. S1. Standard curve for isoamyl ester concentration and peak area.

(A) Isoamyl acetate **(B)** Isoamyl butyrate **(C)** Isoamly hexanoate **(D)** Isoamyl octanoate **(E)** Isoamyl decatnoate **(F)** Isoamyl butyrate (50-500 mM)

J. Microbiol. Biotechnol. 2024. 34(6): 1356–1364
https://doi.org/10.4014/jmb.2402.02033

Table S1. The immobilization yield.

Sample	Protein amount (mg)	Immobilization yield (%) *
Initial solution	31.12	
Supernatant 1	0.90	96.53
Supernatant 2	0.18	

* Immobilization yield = $(P_{initial} – P_{unbound}) / P_{unbound} * 100$
